# Analyze the factors that influence the therapeutic response to psychic trauma

**DOI:** 10.1192/j.eurpsy.2024.1377

**Published:** 2024-08-27

**Authors:** B. Serván Rendón-Luna, G. Strada Herrera, F. Mayor Sanabria, I. López Claramunt, L. Reyes Molón

**Affiliations:** Hospital Clínico San Carlos, Madrid, Spain

## Abstract

**Introduction:**

Psychic trauma profoundly affects a person’s confidence in himself and others. There is a sudden experience of helplessness, loss of control, fear for one’s own life, and the humiliation of having been violated. The victim may run out of internal and external reference elements.

**Objectives:**

Describe the factors that influence the development of Post-traumatic stress disorder after experiencing traumatic experiences.

**Methods:**

Review in the literature of the different factors that influence the subject’s response to the traumatic experience.

**Results:**

1. **Predisposing and precipitating factors:**
  - characteristics of the traumatic evento: severity of the stressor agent: dose-dependent, Characteristics of the same: sudden, prolonged, repetitive, intentional; decrease the ability to control the situation and develop effective coping strategies; they question basic cognitive schemas; the symbolic meaning of the traumatic evento.
  - characteristics of the person (predisposing factors of vulnerability): genetic-constitutional vulnerability, adverse experiences in childhood, previous traumatic events: increased vulnerability, personality characteristics, recent stressors or life changes, inadequate support system, use of alcohol, perception locus control more external than internal, pre-existing psychiatric symptoms: neuroticism, anxiety, depression, critical ages of development: time of greatest vulnerability (11-16 years).
2. **Perpetuating and empowering factors:** sharing traumatic events, seeking the logic of the facts, rupture of affective ties.
3. **Elements of Resistance:** tendency to selectively remember the positive elements in autobiographical memory, acceptance of a certain dose of uncertainty in life, perceiving themselves as survivors, perception of the stressful stimulus as less threatening, Less physiological reactivity to stress, use of humor, positive emotions counteracting during the traumatic process.
4. **Elements of Resilience:** ability to extract and assimilate positive elements from negative situations.

**Conclusions:**

Trauma threatens 3 basic assumptions of life: the world is good, the world has meaning, the self has value. The knowledge of these mentioned factors allow a better psychotherapeutic approach to Psychic Trauma.

**Disclosure of Interest:**

None Declared

